# Bone Marrow-Derived Cells and Wound Age Estimation

**DOI:** 10.3389/fmed.2022.822572

**Published:** 2022-01-27

**Authors:** Yuko Ishida, Mizuho Nosaka, Toshikazu Kondo

**Affiliations:** Department of Forensic Medicine, Wakayama Medical University, Wakayama, Japan

**Keywords:** bone marrow-derived cells, hematopoietic stem cells, mesenchymal stem cells, skin wound, wound healing, wound age

## Abstract

Appropriate technology as well as specific target cells and molecules are key factors for determination of wound vitality or wound age in forensic practice. Wound examination is one of the most important tasks for forensic pathologists and is indispensable to distinguish antemortem wounds from postmortem damage. For vital wounds, estimating the age of the wound is also essential in determining how the wound is associated with the cause of death. We investigated bone marrow-derived cells as promising markers and their potential usefulness in forensic applications. Although examination of a single marker cannot provide high reliability and objectivity in estimating wound age, evaluating the appearance combination of bone marrow-derived cells and the other markers may allow for a more objective and accurate estimation of wound age.

## Introduction

Wound healing is a dynamic process in which numerous cells and extracellular matrix structures are involved. These cellular and molecular events are highly regulated. Wound healing is an ordered and controlled progression that matures through artificially defined phases of hemostasis (coagulation), inflammation (infiltration of granulocytes and mononuclear cells), proliferation (epithelization, fibroplasia, and angiogenesis), and maturation (collagen deposition and formation of scarring tissue) (1–5) (Table 1).

**Table 1:** Wound healing phases.

**Wound healing phases**	**Main events**
Homeostasis	When blood vessels constrict, platelets are activated by contact with exposed collagen, releasing their granules, which further leads to platelet activation and aggregation. Along with activation of the coagulation cascade which results in the deposition of a temporary fibrin matrix within the wound (1, 2).
Inflammation	Numerous cytokines are secreted to promote neutrophil and macrophage chemotaxis, leading to the onset of the inflammatory phase (2, 3). Neutrophils are one of the first cells to appear acutely. Macrophages aid in phagocytosis and produce more cytokines and growth factors that promote fibroblast proliferation, angiogenesis, and keratinocyte migration.
Proliferation	Fibroblasts recruit to the wound transform into myofibroblasts under the influence of several cytokines, causing increased collagen production and eventual wound contraction (4, 5). Modeling and establishment of new blood vessels are important in wound healing and occurs simultaneously at all stages of the repair process (2).
Maturation	Granulation tissue is replaced by permanent scar (2).

In the first step, platelet activation and the coagulation cascade play a major role, with fibrin strands adhering in the first few seconds, subsequently forming blood clots and trapping platelets in the wound area. The inflammatory phase is triggered by the recruitment of inflammatory cells to the wound site and attempts to eliminate damaged cells. Leukocyte recruitment is a hallmark of the inflammatory phase. In the first event, neutrophils infiltrate the wound site for the sterilization, followed by the accumulation of monocytes and lymphocytes. These leukocytes secrete various bioactive molecules, such as cytokines, chemokines, enzymes, and growth factors (6). Cytokines and chemokines are involved in the wound healing process by recruiting leukocytes. To date, several candidates, including IL-1, IL-6, IL-8, and TNF-α, have been identified for wound age determination in the early phase after injury (7–9).

The main objective of the proliferative phase is to cover and fill the wound. The margins of the wound start contacting with fibroblasts that are activated and differentiate into myofibroblasts. Thereafter, the re-epithelialization process also begins. This stage mainly results from extracellular matrix (ECM) deposition of collagen (10–12). Finally, during maturation, collagen fibers are reorganized from collagen type III to type I, tissue is restructured, and strength and flexibility are gained by promoting epithelialization and angiogenesis (13–15). In forensic pathology, growth factors capable of stimulating cell proliferation and cellular differentiation, such as TGF-α and TGF-β1, and some types of collagens have been shown to be available for wound age estimation (16, 17).

Recently, several lines of accumulating studies have shown that bone marrow (BM)-derived cells (BMDCs) may contribute to tissue repair and/or regeneration of damaged tissue including the skin (18–21). After tissue injury, hematopoietic and multipotent progenitor cells are mobilized from the BM into a pool of circulating cells, which migrate to the site of injury and regulate the proliferation and migration of epithelial and dermal mesenchymal cells in the early inflammatory phase (22). The contribution of BMDCs to inflammatory cells in the acute response to injury is well-established, and the long-term role of BMDCs in the healing of skin wounds is being elucidated.

In this review, we assess the characteristics and key functions of BMDCs at each step of the wound healing process and whether they can be useful markers for forensic diagnosis of wound age.

## History of Wound Age Estimation

Raekallio first introduced the application of a new method of enzymatic histochemistry and presented some new data for estimating wound age (23). A few years later, an important biochemical technique was reported that involved the detection of serotonin and histamine at the wound edge (24, 25). Over the next decade, significant progress has been made in the scientific research on immunology and immunohistochemistry. The application of immunohistochemical techniques has paved the way for a new field of wound age research by forensic pathologists (26). In the following decade, knowledge of basic immunological principles and application of immunohistochemical methods have led to significant scientific development (7, 8, 17, 27–44). The history of clinical medicine has been correlated with advances in basic research. Since forensic medicine is applied medicine, it is always necessary to apply the latest basic research knowledge to practice forensic medicine.

## BM-Derived Hematotoietic Stem Cells

### BM-Derived Hematopoietic Stem Cells (BM-HSCs) in Wound Healing

Hematopoietic stem cells (HSCs) constitute a relatively large fraction of BM mononuclear cells (45). Differentiation of HSCs into macrophages is one of the most important events during wound healing (46). There exist two major sources of wound macrophages: resident and BM; the latter accounts for a larger proportion and plays a dominant role in wound healing (47–49). When attracted to the wound, monocytes differentiate into macrophages, which can engage in multiple activities with many possible phenotypes (50, 51). Early in the healing process, macrophages produce multiple cytokines and chemokines that stimulate the inflammatory response (52, 53). Wound macrophages actively phagocytose, removing microbes, dying cells, and necrotic material (54). Several studies have suggested that macrophage phagocytosis of senescent neutrophils causes a switch from a pro-inflammatory to a growth-promoting phenotype (55).

**Figure 1:**
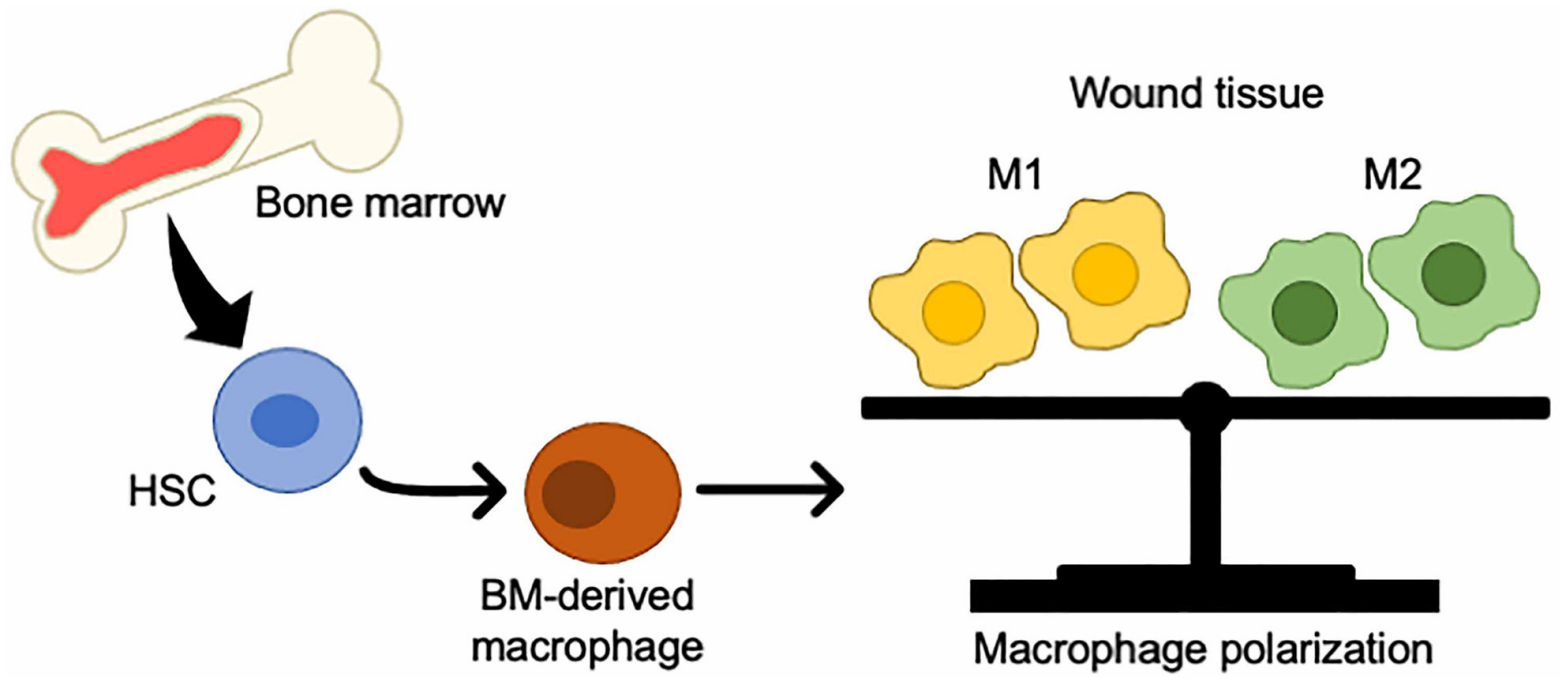
Changes in BMDCs to macrophages in wound tissue. BM-HSCs toward M1/M2 macrophages in injured tissue.

BM-derived monocytes from the circulation are classified as either inflammatory monocytes, which are CD14^+^CD16^−^ that can differentiate into M1 macrophages, or anti-inflammatory monocytes, which are CD14^low^CD16^+^ that give rise to M2 macrophages (56). In a mouse model of wound healing, circulating monocytes can also be divided into two groups: CX3CR1^low^CCR2^+^Ly6C^+^, which produces inflammatory cytokines and enters the wound first, and CX3CR1^high^CCR2^−^Ly6C^−^ which enters later (57). M1 macrophages play an important role in protection from pathogens by producing high levels of iNOS and inflammatory cytokines such as TNF-α, IL-1b, IL-6, and IL-12, and initiate a Th1 immune response (58). M2 macrophages have anti-inflammatory properties and are characterized by high IL-10 secretion and high arginase-1 expression (58, 59) (Figure 1).

The potential of BM-HSCs in skin regeneration is derived from their high plasticity and involvement in the angiogenesis (60, 61). In addition, they also affect ECM during wound healing by secreting collagen and downregulating MMP expression (62). Moreover, they stimulate the proliferation of keratinocytes and fibroblasts, significantly accelerating wound closure (63).

### Macrophages in Wound Age Estimation

Macrophages are mononuclear phagocytes that are recruited from the BM under inflammatory conditions, such as tissue repair (64). Macrophages are involved in host defense, the initiation and resolution of inflammation, growth factor production, phagocytosis, and tissue restoration in wounds (65). During inflammation, macrophages are recruited to the wound site to develop classical and alternative activation phenotypic polarization mediated by cytokines, oxidants, lipids, and growth factors released by macrophages (57, 64, 66). These cells regulate the response to changing wound environments and participate in multiple overlapping wound healing phases.

During early wound healing, macrophages help to clear the wound of contaminating microbes and apoptotic neutrophils and debris via phagocytosis (67–69). In addition, macrophages regulate the activity of other wound cells through the production and release of cytokines, chemokines, and growth factors. Early after injury, macrophages release numerous inflammatory cytokines and chemokines, including IL-1β, IL-6, TNF-α, and CCL2, to amplify the inflammatory response (70). Topical application of CCL2 could promote skin wound healing in diabetic mice, and these effects may be mediated by the action of CCL2 on macrophages (71). Indeed, immunohistochemical studies on the time-dependent expression of chemokines in human skin wounds have shown that inflammatory macrophages are positive for anti-CCL2 antibodies; moreover, a positive rate of > 30% for CCL2 indicates a wound age of at least 1 day (8).

Cyclooxygenase (COX) is an enzyme that is responsible for the formation of prostanoids, including thromboxane and prostaglandins such as prostacyclin, from arachidonic acid (72). COX-1 is constitutively expressed under physiological conditions, and COX-2 is expressed for increased production of prostanoids that occur at the site of disease and inflammation. Therefore, COX-2 may be involved in the inflammatory phase of wound healing. In human skin wound specimens, neutrophils are the main COX-2 expressing cells; however some macrophages also express COX-2 (73). In addition, the number of MMP-2^+^ and MMP-9^+^ macrophages significantly increase with wound age (74). These observations indicate that immunohistochemical detection of increased number of MMP-2^+^ and MMP-9^+^ macrophages in skin wounds, in combination with other markers such as COX-2, further enhances the reliability of wound age estimation.

Wound macrophages are also an important source of growth factors such as VEGF, which is important for angiogenesis (75, 76). Moreover, macrophages have been shown to be involved in collagen degradation during the tissue remodeling phase of wound healing (77, 78). In human wound specimens with wound ages of >7 days, granulation tissue and angiogenesis were observed with the migration of VEGF^+^ macrophages (79).

### Mast Cells in Wound Age Estimation

Mast cells (MCs), that are one of the immune cells involved in allergy and anaphylaxis, play pivotal roles in skin wound healing thorough the release of chemical mediators such as histamine and the production of cytokines and chemokines (80). From the aspects of wound age estimation, there are several immunohistochemical studies on the dynamics of MCs with focusing on triptase and chimase (81, 82). The number of MCs immediately increased after wounding, eventually reaching a peak at 1–3 h later followed by decreasing within 6 h (81, 82). The post-mortem release of proteins from MCs is known as an influence factor on the data interpretation, which should be taken into consideration in the forensic practices (83). In order to avoid the influences of the postmortem release from MCs, the stem cell factor (SCF) and the Kit receptor, which involved in the survival, growth, migration, and activation of MCs, are investigated. Actually, SCF^+^ cells rapidly increased in the dermis by day 1 after injury, whereas the Kit receptor elevated more gradually, with a peak on day 14 (84). On the contrary, Oehmichen et al. (85) investigated the loss of MC enzymatic activity at the wound margin, and found the loss of naphtol AS-D chloroacetate esterase (NASDCAE) activity at wound margins in injuries of <60 min (85).

### Dendritic Cells (DCs) in Wound Age Estimation

DCs are mononuclear and antigen presenting immune cells. DCs have an ultimate origin in HSCs from the BM (86). Intermediate precursors of DCs lack a lineage-specific marker (lin^−^) and can be sought among BM cells that have not yet expressed DC markers such as CD11c and surface MHC class II molecules. Later, DC precursors can be found in BM cells that already express the DC marker CD11c, but that still lack cell surface expression of MHC class II molecules (86).

Several studies have indicated that dermal DC recruitment may be involved in the repair process of damaged tissue (87–90). CD11c and HLA-DR are considered specific markers for dermal DCs (91). Kuninaka et al. performed a double-color immunofluorescence analyses with anti-CD11c and anti-HLA-DRα antibodies to detect DCs in human skin wounds from autopsies (92). DCs were rarely detected in wounds aged <1 day, whereas DC accumulation increased over time in wounds aged 3–14 days. These findings suggest that DCs could be a useful cellular marker for determining wound age.

There is a specific DC population in the human epidermis, and those epidermal DCs express CD1a and CD207/langerin, and is called Langerhans cells (93). However, there is only one forensic study exploring the dynamics of dermal DCs after wounding. Bacci et al. (94) investigated the behavior of epidermal DCs/Langerhans cells in relation with wound ages. Both MHC-II^+^ cells and CD1a^+^ cells rapidly increased in number within the first hour after injury. Especially, CD1a^+^ cells, as well-differentiated Langerhans cells, increased earlier and for a shorter time period than MHC-II^+^ cells. These observations implied that the behavior of epidermal DCs/Langerhans could give a useful information to differentiate antemortem skin lesions from postmortem damage especially in neck compression cases.

## BM-Derived Mesenchymal Stem Cells

### History of BM-Derived Mesenchymal Stem Cells (BM-MSCs)

BM contains HSCs and MSCs. MSCs were first observed in the BM by Cohnheim in 1867 (95). Cohnheim discovered that these cells could be a source of fibroblasts involved in wound repair. Subsequently, these cells were isolated and cultured by Friedenstein (96). While culturing cells from rat BM, Friedenstein discovered that these cells were a population of non-hematopoietic cells that were morphologically similar to fibroblasts attached to the plastic of the culture flask. The term “mesenchymal stem cells” was presented by Caplan in 1991 after conducting human BM studies (97). To date, it is a hot topic of research that is being explored for multiple purposes.

### BM-MSCs in Wound Healing

With the expansion of MSC research, its potential role in skin wound healing has been elucidated. BM-MSCs can accelerate wound healing by regulating the function of inflammatory cells such as neutrophils, macrophages and lymphocytes to provoke an anti-inflammatory response (98). In addition, BM-MSCs can be directed to differentiate into multiple skin cell lineages, including keratinocytes and endothelial cells, and secrete various cytokines to promote wound re-epithelialization and limit excessive scarring (98–103). In addition, BM-MSCs can be recruited to the wound site to induce neovascularization and to increase cell migration and proliferation (104, 105).

Several studies have revealed the underlying mechanisms of BM-MSC recruitment to the wounds. BM-MSCs express CCR7, a receptor of CCL21, which was found to be the main factor responsible for enhanced BM-MSC migration to the wounds in mice (106). Intradermal injection of CCL21 increased the recruitment of BM-MSCs to the wound, resulting in accelerated repair (106). Moreover, serum levels of HMGB1 are increased by skin grafting, and intravenously administered HMGB1 augment the accumulation of PDGFRα^+^ MSCs in the skin graft by enhancing the expression of the SDF-1 receptor CXCR4 in these cells (107, 108).

The inflammatory phase is important for the wound healing process because it leads to the recruitment of immune cells to remove pathogens and clear the wound. MSCs can suppress the inflammatory responses in several ways. It is generally recognized that infiltrative M2 macrophages play an important role in the progression of wound healing, the promotion of angiogenesis, and the suppression of inflammation (109–111). MSCs promote macrophage polarization to the M2-like functional phenotype, which reduces inflammation and immunosuppressive function (112). Zhao et al. revealed that IL1RA from BM-derived MCSs inhibits the production and activity of IL-1 and TNF-α (113). These studies suggest that MSCs exhibit anti-inflammatory potential through the regulation of macrophage polarization and expression of anti-inflammatory cytokines.

In the proliferative phase, macrophages release growth factors such as EGF and TGF-α to stimulate keratinocyte migration and proliferation (114). Smith et al. revealed that BM-MSCs are a source of soluble signals that regulate dermal fibroblast migration and proliferation (115). MSCs can also contribute to angiogenesis at the wound site. In the wound area, MSCs secrete growth factors such as VEGF, PDGF, bFGF, and angiopoietin-1 to promote angiogenesis and wound healing (116, 117). In addition, SDF-1 secreted by MSCs induces endothelial cell survival, vascular branching, and pericyte recruitment (118). These paracrine mechanisms of MSCs play important roles in angiogenesis. The wounds treated with MSC-seeded hydrogels showed a significant enhancement of angiogenesis, which was associated with elevated VEGF levels within the wound (119). Qiu et al. demonstrated that educated MSC exosomes significantly increase wound healing by inducing angiogenesis (120).

MSCs have also been shown to contribute to the production and remodeling of ECM during the wound healing process. BM-MSCs secrete high levels of TIMPs, which stabilize vessels and protect the vascular basement membrane, forming MMP-induced degradation (121). This ECM production and remodeling function of MSCs may be associated with the promotion of angiogenesis and the formation of granulation tissue.

BM-MSCs may be involved in the regeneration of mesenchymal and other embryonic tissues, including the skin (106). In animal models of wound healing, intravenously transplanted MSCs can differentiate into cells of resident tissue, including fibroblasts, myofibroblasts, vascular endothelial cells, pericytes, and keratinocytes in the wound area (106, 116). In addition, MSCs injected into mouse wounds transdifferentiate into keratin-14^+^ keratinocytes *in vivo* (106, 116). Labeled MSCs were observed in the hair follicles, sebaceous glands, and blood vessels in full-thickness wounds in an animal model (122). BM-MSC-engineered skin (EGF loaded) has been found to repair sweat glands and improve skin wound healing (123).

**Figure 2:**
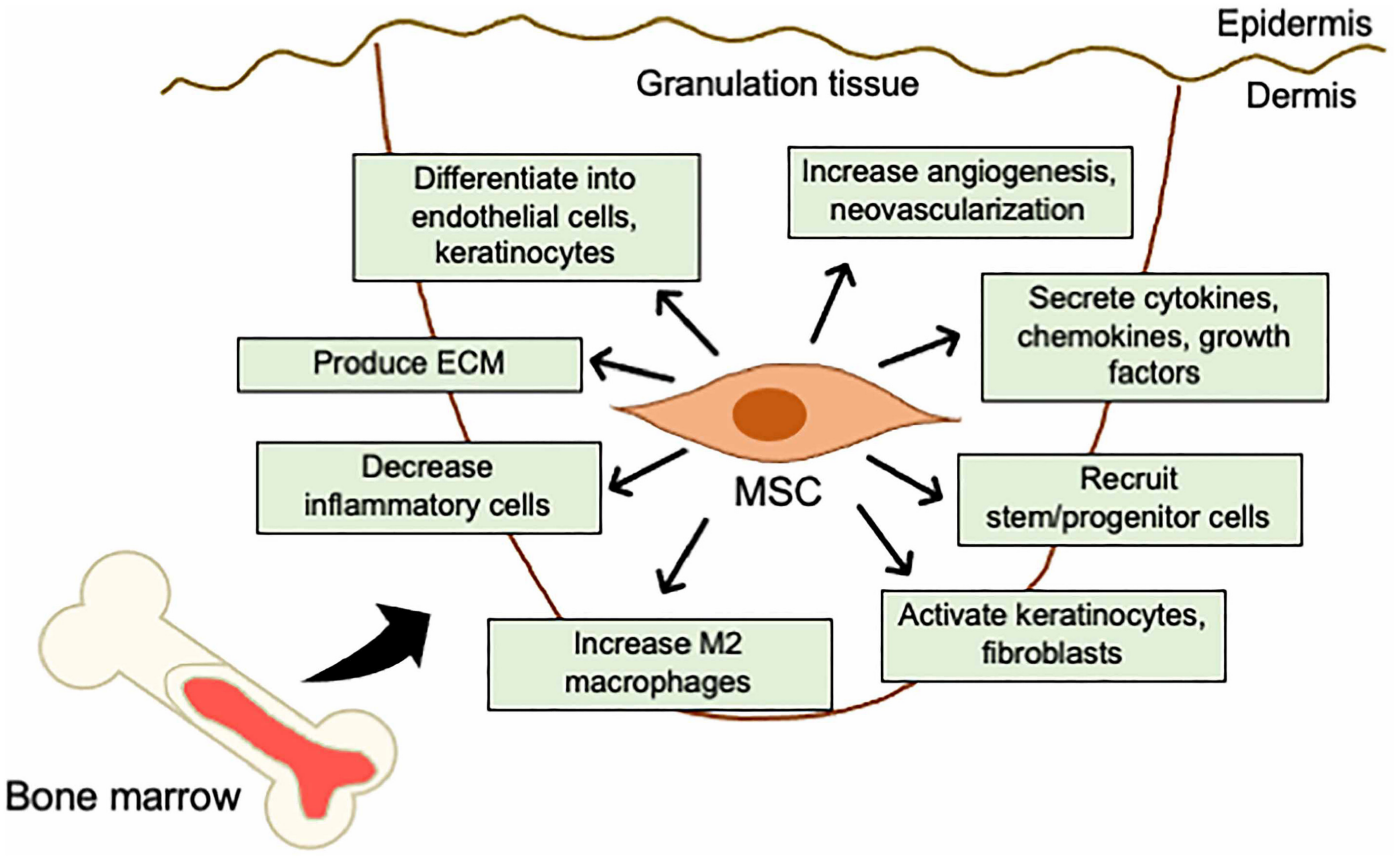
Mechanistic roles of MSCs in the skin wound healing. Mechanisms of acceleration of wound healing by MSCs; (i) activation of keratinocytes and fibroblasts, (ii) increase in angiogenesis and neovascularization, (iii) increase in M2 macrophages infiltration, (iv) recruitment of stem/progenitor cells, (v) secretion of cytokines and growth factors, (vi) production of ECM, (vii) decrease in inflammatory cytokine levels by immunosuppressive effects, and (viii) differentiation into endothelial cells, fibroblasts, and keratinocytes.

These studies indicate that BM-derived MSCs can differentiate into tissue-specific cells, secrete a wide range of paracrine factors, and regulate the immune response and the local tissue microenvironment (Figure 2).

### Fibrocytes in Wound Age Estimation

In 1994, a distinct population of blood-borne fibroblast-like cells that rapidly entered sites of tissue injury was described (124). These cells, named “fibrocytes,” comprise 0.1–0.5% of the non-erythrocyte cells in the peripheral blood and show an adherent, spindle-shaped morphology when cultured *in vitro*. Cultured fibrocytes express the fibroblast products including type I collagen I (Col I), type III collagen (Col III), and fibronectin, CD45RO, CD13, and CD34. Additionally, fibrocytes express MHC class II and costimulatory molecules (CD80 and CD86) and can present antigens *in vitro* and *in vivo* (125, 126). Fibrocytes differ from monocytes/macrophages, dendritic cells, and other antigen-presenting cells in their morphology, growth properties, and cell surface markers. In addition, fibrocytes isolated from peripheral blood and cultured *ex vivo* secrete cytokines, growth factors, and chemokines (127). TGF-β functions as a fibrocyte maturation factor during differentiation (128, 129).

There is an increasing evidence that fibrocytes contribute to new fibroblast and myofibroblast populations during wound healing. Prior to differentiation, immature fibrocytes secrete ECM-degrading enzymes, including MMP-2, -7, -8, and -9 which promote the migration of fibrocytes into granulation tissue and endothelial cell invasion (130, 131). CCL21 acts as a potent stimulus for fibrocyte chemotaxis *in vitro* and for the migration of injected fibrocytes to sites of skin wound site *in vivo* (128). In addition, exogenous TGF-β1 stimulates *in vitro* differentiation and synthetic activity of cultured human fibrocytes into mature fibroblasts or myofibroblasts (128). Moreover, fibrocyte differentiation can occur in conditions where serum amyloid P (SAP) and aggregated IgG levels are low, such as during the resolution phase of inflammation (132, 133). The main fibrocyte secreting cytokines include TGF-β1 and CTGF (134). Moreover, fibrocytes indirectly regulate resident fibroblast activity during wound healing (132).

Although the role of fibrocytes in wound healing has been postulated based on their accumulation at the wound sites (124), the molecular signals that mediate the migration of fibrocytes to the wounds have not been investigated. Abe et al. demonstrated that fibrocytes express several chemokine receptors, such as CCR3, CCR5, CCR7, and CXCR4 (128). Furthermore, Ishida et al. showed that Ccl3^−/−^ and Ccr5^−/−^ mice exhibit reduced bleomycin (BLM)-induced fibrosis and the number of CCR5^+^ fibrocytes in the lungs compared to wild-type mice (135). This finding indicates that the CCL3-CCR5 axis can mediate the migration of BLM-induced fibrocyte to the lungs. In addition, fibrocytes also express CX3CR1, and their population increases in the lungs of mice with BLM-induced pulmonary fibrosis (136). These findings suggests that the CX3CL1-CX3CR1 axis is essential for the development of BLM-induced pulmonary fibrosis by regulating fibrocytes capable of exerting fibrosis-promoting activity. Therefore, some chemokine systems may be involved in the migration of fibrocytes to damaged and fibrotic tissues.

Ishida et al. performed a double-color immunofluorescence analyses using anti-CD45 and anti-Col I antibodies to examine the time-dependent appearance of fibrocytes in human skin wounds of different age groups (137). The appearance of fibrocytes in human skin wounds occurs at least a 4-days post infliction; therefore, detection of fibrocytes could be a useful marker for wound age determination.

### Endothelial Progenitor Cells (EPCs) in Wound Age Estimation

EPCs are cells that act as endothelial precursors and help promote angiogenesis to improve tissue perfusion. EPCs were first described in 1997 as a population of postnatal mononuclear blood cells that have been shown to promote angiogenesis following recruitment from the BM (138, 139). EPCs are positive for the following cell surface markers: CD31, CD45, CD14, CD105, CD146, VEGFR-2, CD144, and von Willebrand factor (vWF). Morphologically they appear spindle-shaped (140, 141), and the presence of CD14 and CD45 on these cells indicates that they are hematopoietic rather than of endothelial origin. In addition, markers such as CD31, CD144, VEGFR-2, vWF, and eNOS are not necessarily endothelium-specific (142). There is no single marker that defines EPCs, and a combination of markers has been used to identify them within a heterogeneous population. EPCs are mobilized from the BM through a complex process involving enzymes, growth factors and cell surface receptors. The first step in the EPC mobilization is MMP-9 activation (143). VEGF plays an important role in the activation of MMP-9 and can increase the recruitment of EPCs from the BM (144). Interestingly, fibrocytes can produce angiogenic factors, including MMP-9 and VEGF, as demonstrated *in vivo* (131).

EPCs are known to be sensitive to hypoxia because they respond to HIF-1-induced SDF-1 under conditions of oxygen deprivation (145). They contribute to angiogenesis and are promising targets for the treatment of chronic wounds such as diabetic ulcers (146–148). Transplanted BM-MSCs induce the recruitment of endogenous EPCs to the wound site from the BM or circulation via growth factors such as VEGF, PDGF, HGF, and insulin-like growth factor and the SDF-1-CXCR4 axis (149–153). Transplantation of human EPCs into a mouse skin wound model has been shown to accelerate wound closure and increase angiogenesis (154). EPC transplantation accelerated wound re-epithelialization in a mouse skin excision wound model compared to that in control mice (155). EPCs produce several chemoattractants of monocytes and macrophages which are known to play important roles in the early stages of wound healing. In addition, EPCs migrate to the wound and are incorporated directly into the newly formed capillaries in the granulation tissue (155). Thus, EPCs have the potential to differente into the endothelium, recruit other cells to the wound site, and secrete growth factors and cytokines; these factors explain the effects on wound healing.

Ishida et al. demonstrated that topical application of CCL2, a potent macrophage chemoattractant, can promote neovascularization, collagen accumulation, and eventual cutaneous wound healing in mice with diabetes (71). The effects of CCL2 may be mediated by EPCs and macrophages, which are critically involved in angiogenesis and collagen production, respectively, and their effects on steps essential to the wound healing process. In addition, the CCL5-CCR5 axis is essential for EPC recruitment (156). In a mouse model of skin wounds, gene expression of the *Ccl5* and *Ccr5* genes was upregulated at the wound sites and CCR5 protein was detected in endothelial cells. *Ccr5*^−/−^ mice showed delayed wound healing with diminished neovascularization. The CCR5^+^ EPCs were directly incorporated into the vasculature at the wound sites. Moreover, EPCs produce growth factors such as TGF-β and VEGF, which are important for skin wound healing (157, 158). These observations suggest that EPCs may contribute to skin wound healing as a source of endothelial cell origin and growth factors.

The accumulation of EPCs in wound sites increases over time after injury; this finding indicates that EPC accumulation may help estimate wound age (156, 159). In forensic practice, examining only a single marker does not provide forensic safety; therefore, some markers need to be investigated in wound samples for a more accurate estimation of wound age. For example, detection of both EPCs and VEGF (79) provides more reliable information for estimating wound age, especially during the proliferative phase, as their collaboration synergistically promotes angiogenesis.

## Conclusion and Future Perspectives

Over the last few decades, numerous studies have elucidated the role of BMDCs in skin wound healing (Table 2). It is clear that BMDCs have great potential for skin tissue regeneration as they not only regenerate lost tissue, but also promote wound repair in a paracrine manner. Several cell types, including HSCs and MSCs, are currently being investigated. Recent data on BM cell therapy in skin repair show great promise as therapeutic agents in clinical practice. Further investigation into experimental and clinical applications is required to identify the most effective cell migration system for BM cells at the wound sites. However, it is evident that BMDCs contribute to skin wound healing; therefore, these cells can serve as candidates for wound age estimation in forensic practice. New molecular biomarkers and innovative devices and technologies are constantly being sought to correctly diagnose the cause of death, postmortem interval, wound age, and more.

**Table 2:** Summary of related studies on the appearance and effects of BMDCs on the skin wound healing process.

**Cell types**	**Markers**	**Methods**	**Model source used**	**Functions, effects/findings**	**Regular detection**	**Time frame**	**References**
Macrophages	F4/80^+^CD115^+^ CD11b^+^	Create a full-thickness wound on the back skin of WT mice, and transplant WT or db/db HSCs	Mouse	FACS analysis show that type 2 diabetes impairs monocytes/macrophages infiltration and ultimately impairs wound healing	3–14 d	3–14 d	(46)
Macrophages	CX3CR1-GFP	Create full-thickness wounds on the back skin	Mouse	FACS analysis show the GFP^hi^ population increases after injury	4–7 d	0–7 d	(47)
CD34^+^ stem cells	CD34^+^	Create full-thickness wounds on the back skin and transplant nanofiber-expanded human umbilical cord blood-derived (NEHUCB) CD34^+^ cells	Mouse	GFP-NEHUCB CD34^+^ cells home to wound area and accelerate wound healing	3 h−7 d	3 h to 7 d	(62)
Macrophages	Macrophage morphology	A subcutaneously implanted polyvinyl alcohol (PVA) sponge wound model (7 cm skin incision on the back)	Rat	Phagocytosis of wound macrophages on wound neutrophils	5–10 d	1–10 d	(67)
Macrophages	F4/80^+^	Create full-thickness wounds on the back skin	Mouse	Immunohistochemistry staining of wounds shows that there is no difference in macrophage recruitment to the wounds of WT and PPARγ^−/−^ mice	3–5 d	3–5 d	(69)
Macrophages	F4/80^+^	Create full-thickness wounds on the back skin	Mouse	Western blotting analysis shows that diabetic mice exhibit reduced infiltration of macrophages into wounds, and ultimately impaired healing	Uninjured and days 1–10	Uninjured and days 1–10	(71)
MMP-2^+^ macrophages	CD68^+^MMP-2^+^	Double-color immunofluorescent staining using skin wounds	Human	MMP-2^+^ macrophages on skin wounds are useful markers for determining the age of wounds	9–12 d	Uninjured and 12 h to 21 d	(74)
Macrophages	F4/80^+^CD11b^+^	Create full-thickness wounds on the back skin	Mouse	FACS analysis shows that CCR2 deficiency reduces macrophage infiltration into the skin wounds	2–7 d	2–14 d	(76)
DCs	FXIIIa^+^	Immunostaining burn specimens	Human	Need further studies to clarify the significance of FXIIIa expression by dermal cells	Uninjured and days 5–30		(87)
Plasmacytoid DCs (pDCs)	PDCA1^+^B220^+^	Measure pDCs in tape stripped skin by flow cytometry	Mouse	Immunohistochemistry for Siglec-H, pDC-specific marker, shows lymphocytic cells in injured skin	24 h	24–48 h	(89)
pDCs	BDCA2^+^	Immunostaining tape stripped skin	Human	Injury induces pDC infiltration and expression of IFN-α	24 h		(89)
DCs	CD11C^+^MHC-II^+^Ly6G^−^	Measure DCs in burned skin by flow cytometry	Mouse	Wound closure in DC-deficient mice is delayed	4 d		(90)
DCs	CD11c^+^HLA-DRα^+^	Double-color immunofluorescent staining using skin wounds	Human	The appearance of DC in human skin wounds provides information to help determine the age of the wound	4–14 d	3–21 d	(92)
MSCs	GFP^+^	Create a full-thickness excisional skin wound and transplant with GFP^+^ MSCs	Mouse	FACS analysis show that about 10% of total cells in day 7 wounds are GFP^+^ BM-MSC, and MSCs enhance wound healing	7–14 d	7–28 d	(116)
Fibrocytes	Col I^+^CD34^+^	Implant the wound chamber	Mouse	10–15% of the cells present in wound chamber fluid are fibrocytes	Rapidly	Over 10 d	(124)
Fibrocytes	Col I^+^CD11b^+^	Inject cultured murine fibrocytes into the tail vein and create a full-thickness skin wound	Mouse	Chemokine SLC acts as a potent stimulus for homing of fibrocytes to the site of tissue injury	4 d		(128)
Fibrocytes	Col I^+^	Culturing peripheral blood mononuclear cells (PBMC) in burn patients	Human	Fibrocyte development is systemically increased in burn patients	7 d to 12 m	7 d to 12 m	(129)
Fibrocytes	CD45^+^Col I^+^	Double-color immunofluorescent staining using skin wounds	Human	Fibrocytes are involved in wound healing in human skin, and detection of fibrocytes is a useful marker for wound age determination	9–14 d	4 d to 21 d	(137)
Human EPCs	acLDL^+^ulex-lectin^+^	EPC transplantation into a dermal excisional wound model	Mouse	EPC transplantation increases neovascularization and ultimately accelerates wound re-epithelialization			(1)
Mouse EPCs	c-Kit^+^Tie-2^+^	Create full-thickness wounds on the back skin	Mouse	FACS analysis shows that the absence of CCR5 reduce vascular EPC accumulation, and ultimately delay skin wound healing	2–4 d	2–4 d	(156)
Human EPCs	CD34^+^Flk-1^+^	Double-color immunofluorescent staining using skin wounds	Human	EPC detection helps determine wound age	7–12 d	2–21 d	(159)

Recent studies have stimulated us to recognize the importance of BMDCs in the skin; however, many questions remain. For example, BMDCs contribute not only to inflammatory and mesenchymal cells of the dermis, but also to keratinocytes of the epidermis. In addition, it is not yet known whether BM-derived cells are essential to contribute to the cells that make up the normal skin. Furthermore, the specific types of BMDCs playing a role in these processes are unidentified. We believe that answers to these questions will help us understand skin homeostasis and the wound healing process as well as develop new techniques for future skin wound age estimation in future.

Finally, there is a limitation of wound age estimation as the forensic evidence. From the aspects of forensic pathology, the purpose of wound age estimation is to present the objective evidence in court. Cell types, enzymes and chemical mediators are experimentally and practically applied to wound age estimation as the marker (160). Actually, when only a single marker is investigated, contradictory results are often obtained, eventually making confusion the interpretation of data. It is needless to say that various populations of BMCs and BMC-derived enzymes as well as chemical mediators should be investigated. Moreover, among multiple forensic institutes, the accumulation of practical evidence using different types and sizes of wound samples with known post-injured intervals is necessary.

## Author Contributions

YI and TK contributed to conception and design of the review, drafted the original manuscript and drew the figures, and provided the main funding for the manuscript. MN edited various versions of the manuscript. All authors read and approved the final version of the review.

## Funding

This study was financially supported in part by Grants-in-Aid for Scientific Research (B, 20H03957, YI) and (B, 18H03067, TK) from JSPS.

## Conflict of Interest

The authors declare that the research was conducted in the absence of any commercial or financial relationships that could be construed as a potential conflict of interest.

## Publisher's Note

All claims expressed in this article are solely those of the authors and do not necessarily represent those of their affiliated organizations, or those of the publisher, the editors and the reviewers. Any product that may be evaluated in this article, or claim that may be made by its manufacturer, is not guaranteed or endorsed by the publisher.
